# Chemotaxis promotes early colonization and microbiogeography of *Caballeronia insecticola* in the gut symbiotic organ of *Riptortus pedestris*

**DOI:** 10.1128/aem.01364-26

**Published:** 2026-09-14

**Authors:** Kota Ishigami, Antoine-Olivier Lirette, Hiroyuki Shimoji, Yoshitomo Kikuchi

**Affiliations:** 1 Faculty of Agriculture, University of the Ryukyus 26428, Ginowan, Okinawa, Japan; 2 Biomanufacturing Process Research Center, National Institute of Advanced Industrial Science and Technology (AIST) 13508, Sapporo, Japan; 3 Graduate School of Agriculture, Hokkaido University 12810, Sapporo, Japan; 4 Department of Environmental Science and Conservation Biology, The United Graduate School of Agricultural Sciences, Kagoshima University 12851, Kagoshima, Kagoshima, Japan; Norwegian University of Life Sciences, Ås, Norway

**Keywords:** symbiosis, *Caballeronia*, chemotaxis

## Abstract

Many plants and animals form specific symbioses with microorganisms, relying on bidirectional host–bacteria interactions. However, knowledge about the evolution of symbiont traits enabling such specificity remains limited. The bean bug *Riptortus pedestris* acquires *Caballeronia* from environmental soil and harbors it in its gut symbiotic organ. This bug-*Caballeronia* symbiosis is an ideal model to clarify the evolutionary process of symbiotic bacteria because members of outgroups, such as *Paraburkholderia* and *Pandoraea*, can also colonize the host symbiotic organ but are outcompeted when co-inoculated with the native symbiont, *Caballeronia*. Here, we investigated mechanisms underlying the competitive performance of *Caballeronia* within the insect gut. Spatial analyses revealed that wild-type *Caballeronia* showed directional localization toward the symbiotic organ and colonized the M4 region faster than related bacteria. A *cheA* insertion mutant exhibited delayed early colonization and reduced competitive performance in co-infection assays although both strains ultimately reached similar levels of crypt colonization. In addition, microscopic observations revealed strain-specific aggregation patterns within the symbiotic organ, providing additional insight into bacterial spatial organization during host colonization. Together, these findings suggest that chemotaxis accelerates early symbiotic organ colonization and enhances competitive performance during the initial stages of symbiont establishment. More broadly, our results highlight the importance of spatial and temporal dynamics in shaping host–microbe associations and provide a framework for understanding how bacterial traits contribute to the evolution of symbiotic specificity.

**IMPORTANCE:**

*Riptortus pedestris*, a major soybean pest in East Asia, acquires symbiotic bacteria from the environment every generation, yet its gut is consistently and specifically colonized by *Caballeronia* species. The evolutionary traits that underlie this strong symbiotic specificity remain poorly understood. Here, we demonstrate that chemotaxis accelerates early colonization of the symbiotic organ by *Caballeronia* and enhances its competitive performance relative to closely related bacteria. Using comparative colonization assays with wild-type, chemotaxis-deficient mutants, closely related bacteria, and an out-group species, we show that chemotaxis contributes to competitive performance during the initial stages of symbiotic organ colonization. Our findings indicate that ecological and behavioral traits, including chemotaxis, contribute to the establishment of exclusive symbiotic associations, providing new insight into how symbiotic specificity may evolve in horizontally transmitted systems.

## INTRODUCTION

Most plants and animals form symbiotic relationships with microorganisms. Some symbioses establish specific and exclusive relationships. In such symbiosis, hosts have developed sophisticated partner choice mechanisms to acquire particular bacterial symbionts (1, 2). The symbioses achieve partner specificity through various host mechanisms (1–4), such as signal recognition and the secretion of antimicrobial peptides (5, 6). This specificity is not achieved solely by host traits; symbionts must also possess corresponding abilities to establish the relationship. In essence, specific symbiotic relationships are formed through bidirectional interactions rather than unidirectional processes. However, evolutionary insights into the abilities of symbiotic bacteria to establish specific symbiosis remain very limited. This limitation arises because systems with highly specialized symbiotic relationships often cannot associate with a wide variety of bacterial partners, including outgroup strains. As a result, it is experimentally difficult to investigate the stepwise processes by which such highly specific and stable partnerships have evolved.

**Fig 1:**
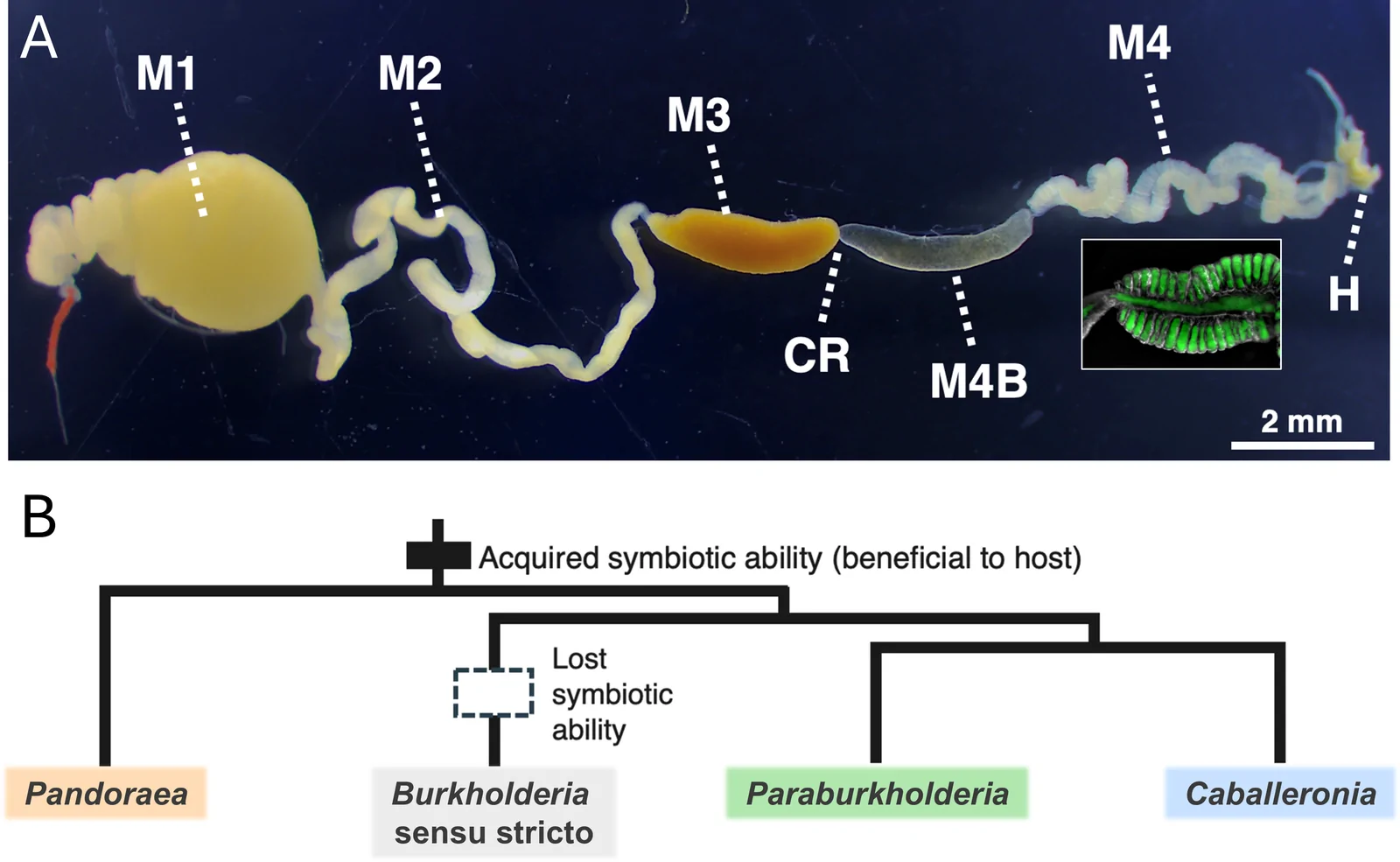
Anatomical structure of the symbiotic organ and phylogenetic context of symbiotic *Burkholderia sensu lato*. (**A**) Dissected midgut of *Riptortus pedestris* infected with *Caballeronia insecticola*: M1, midgut first section; M2, midgut second section; M3, midgut third section; CR, constricted region; M4B, bulbous region prior to M4; M4, crypt-bearing midgut fourth section; H, hindgut. An enlarged image of the M4 crypt-bearing region is shown within the white box. The crypt-bearing region consists of bilaterally symmetric pairs of crypts arranged along the anterior–posterior axis. GFP fluorescence (green) indicates bacterial localization within the crypt lumen. (**B**) Dendrogram of the phylogenetic relationships between *Pandoraea norimbergensis* and genera of *Burkholderia sensu lato*. Note that all genera except *Burkholderia* sensu stricto are capable of colonizing the M4 region. The black box represents the evolution of symbiotic ability, and the white box represents the loss of symbiotic ability.

The bean bug *Riptortus pedestris* provides an ideal model for studying the evolution of traits in symbionts for establishing specific symbiotic relationships. The stink bug is widely distributed in Southeast Asia and has a midgut divided into four sections, M1 to M4 (4). The M1 to M3 are involved in digestion and infected with diverse microbial communities, while M4 is colonized by a stable, high-density population of a single microbial symbiotic species of the genus *Caballeronia* (Fig. 1A) (7–9). In the M4 region, there are sac-like structures called crypts, densely packed with symbiotic bacteria. Although these symbionts are not essential for the survival of *R. pedestris*, they benefit the host insect by enhancing growth, reproduction, immune homeostasis, and pesticide resistance (6–8, 10–12). *Riptortus pedestris* acquires microbial partners from environmental soil each generation (8, 13). Despite the presence of diverse bacteria in the environmental soil, *R. pedestris* specifically retains *Caballeronia* bacteria in its symbiotic organ.

Environmental bacteria ingested by *R. pedestris* are strictly selected by the “constricted region (CR)” located between M3 and M4, the lumen of which is only a few micrometers in diameter and is filled with mucus-like viscous substances. *Caballeronia* symbionts are capable of crossing the CR, whereas phylogenetically distant bacteria such as *Escherichia coli* cannot. Following infection by symbiotic bacteria, the CR region completely closes, preventing secondary infection (14, 15). Additionally, upon bacteria reaching the symbiotic organ, various antimicrobial peptides are specifically expressed, contributing to host specificity (6). Notably, while *R. pedestris* has these multiple mechanisms for partner choice, bacterial groups closely related to the genus *Caballeronia*, such as *Paraburkholderia* from the *Burkholderia sensu lato* group and *Pandoraea* from the out-group of *Burkholderia sensu lato*, can also pass through the sorting organ (CR), reach the symbiotic organ (M4), and proliferate and establish colonization in the symbiotic organ similar to *Caballeronia* (Fig. 1B) (16). However, previous studies have shown that when *Pandoraea norimbergensis* or *Paraburkholderia fungorum* are orally inoculated simultaneously with *Caballeronia*, the latter outcompetes the formers, ultimately dominating the symbiotic organ (16). What causes such a difference in the infectivity between the closely related bacterial groups remains unclear.

In this study, we closely observed the initial infection process of three bacteria: *Caballeronia insecticola*, *Paraburkholderia fungorum,* and *Pandoraea norimbergensis*, and elucidated quantitative differences in infectivity, such as the time taken to reach the M4 symbiotic organ and the invasion rate into the lumen of M4 crypts. Detailed observations revealed that *Caballeronia insecticola* reaches and passes through the CR faster than other bacteria, enabling earlier colonization and proliferation in the M4 symbiotic organ, suggesting that chemotaxis contributes to early colonization dynamics. In addition, *Pandoraea norimbergensis* and *Paraburkholderia fungorum* exhibited delayed infection and colonization compared with *Caballeronia insecticola*. They also displayed distinct aggregation patterns within the midgut. Together, these findings highlight the importance of early spatial and temporal dynamics in shaping competitive outcomes during symbiotic organ colonization and provide new insight into the evolution of environmentally acquired symbioses.

## MATERIALS AND METHODS

### Insects and bacterial strains

The *Riptortus pedestris* strain used in this study was originally collected from a soybean (*Glycine max*) field in Tsukuba, Ibaraki Prefecture, Japan, and has been maintained in the laboratory for over 10 years. The insects were reared in Petri dishes (90 mm diameter, 20 mm height) at 25°C under long-day conditions (16 h light, 8 h dark) and were provided with soybean seeds and distilled water containing 0.05% ascorbic acid (DWA). For the experiments, GFP-labeled strains of *Caballeronia insecticola*, *Paraburkholderia fungorum*, and *Pandoraea norimbergensis* were used. In addition, a chemotaxis-deficient *cheA* insertion mutant of *C. insecticola* was employed. The *cheA* mutant used in this study was originally generated by Ohbayashi et al. (9) through random mini-Tn5 insertion mutagenesis. Briefly, a hyperactive Tn5 transposon (pRL27) was introduced into *C. insecticola* by conjugation, and transconjugants were screened for altered motility on semisolid agar plates. The *cheA* mutant was originally isolated as a motility-altered mutant and was confirmed in the previous study to carry a Tn5 insertion in the *cheA* gene. The *cheA* gene encodes a histidine kinase that plays a central role in bacterial chemotaxis signaling (17). The *cheA* mutant was labeled with GFP using the Tn7 mini-transposon system as previously described (7). All bacterial strains used in this study are listed in Table S1.

### Oral administration of bacteria

Each symbiotic bacterial strain was cultured at 30°C in YG medium (with 30 μg/mL kanamycin for GFP-labeled strains) on a rotary shaker (150 rpm) until the early logarithmic growth phase. The bacteria were harvested by centrifugation, resuspended in DWA, and adjusted to a concentration of 10^4^ cells/μL. After the first instar nymphs molted into the second instar, DWA was removed from the rearing containers to prevent the nymphs from drinking water overnight. Then, 1 μL of the adjusted bacterial suspension was provided to each second instar nymph, and individuals that consumed the suspension within 1 h were used in subsequent experiments. After infection, the insects were supplied with DWA and reared.

### Microscopic analysis of bacterial localization in the M3 region

To examine the spatial distribution of bacteria within the M3 region prior to entry into the symbiotic organ, dissected midguts were observed at 12, 16, and 20 h after oral inoculation with GFP-labeled bacteria. Insects that orally inoculated bacteria were dissected in phosphate-buffered saline (PBS: 137 mM NaCl, 8.1 mM Na2HPO4, 2.7 mM KCl, 1.5 mM KH2PO4 [pH 7.4]) using tweezers under a dissecting microscope (S8APO, Leica, MZ FZ III, Leica). For each bacterial strain and each time point, five independent insects were analyzed (*n* = 5). The dissected M3 regions were placed on glass slides in PBS and imaged directly under a fluorescence stereomicroscope (M205 FA, Leica, Germany). Images were acquired using identical exposure settings for all samples.

Fluorescence images were processed using ImageJ (NIH). To facilitate visualization of the spatial distribution of GFP signals, fluorescence intensity within each image was converted into a three-dimensional surface plot using the “3D Surface Plot” function of ImageJ. In these plots, fluorescence intensity is represented as height, enabling visual assessment of whether bacterial signals were uniformly distributed or concentrated toward the symbiotic-organ side of the M3 region.

Fluorescence images of the M3 region were processed and analyzed using ImageJ (NIH). Prior to quantification, background fluorescence was subtracted using the rolling ball algorithm with a radius of approximately 80 µm. The same background subtraction parameter was applied uniformly to all images. Following background correction, a linear region of interest was drawn from the oral side toward the symbiotic organ (CR) side in each image. Fluorescence intensity profiles were extracted using the Plot Profile function in ImageJ (Fig. S1). Distance values and corresponding gray values were exported and analyzed in R. To standardize orientation across samples, all profiles were defined such that 0 corresponded to the oral side and 1 corresponded to the symbiotic organ side. Distances were normalized to a 0–1 scale by dividing each position by the maximum profile length. Spatial distribution was quantified by calculating the center of mass (COM) of fluorescence intensity:


$$COM=\frac{\sum (x_{i}\cdot J_{i})}{\sum J_{i}}$$

where *x_i_* is the normalized position and *J_i_* is the background-corrected fluorescence intensity at position *i*. COM values greater than 0.5 indicate enrichment toward the symbiotic organ side, whereas values less than 0.5 indicate enrichment toward the oral side. Each image was treated as an independent replicate. Statistical analyses were performed in GraphPad Prism 10.1.0 (GraphPad Software). For each strain and time point, one-sample *t*-tests were conducted to compare COM values against a theoretical mean of 0.5, representing a uniform distribution along the oral–symbiotic organ axis. All tests were two-tailed. A *P*-value < 0.05 was considered statistically significant.

### Observation of infection dynamics to the symbiotic organ

Insects that orally ingested bacteria were dissected in PBS. The midgut was then observed on a glass slide with PBS using a stereomicroscope, and photographs of the dissected tissues were taken with a digital camera (EC3, Leica). To examine the speed at which symbiotic bacteria reach the symbiotic organ, insects were dissected at regular intervals (0 h, 4 h, 8 h, 12 h, 16 h, 20 h, 24 h, and 40 h) after oral inoculation, and GFP signals in the symbiotic organ (M4B and M4) were observed. At each time point and for each bacterial strain, 20 independent insects were examined (i.e., *n* = 20 per time point). The presence of bacteria in the symbiotic organ was considered positive if at least one GFP-expressing bacterial cell was detected. Infection rates were calculated as the proportion of positive individuals among the 20 examined insects at each time point. To visualize bacterial localization and aggregation patterns within the main duct of the M4 region, symbiotic organs dissected at 40 h post-inoculation were additionally observed using a fluorescence microscope (BX53, Olympus). Images were acquired from the same dissected samples described above under GFP fluorescence. The acquired data were statistically analyzed using the GraphPad Prism 10.1.0 (GraphPad Software). To compare the infection dynamics among bacterial strains, statistical analyses were performed separately at each time point. For each time point, all pairwise comparisons among the four bacterial strains (six combinations) were conducted using Fisher’s exact tests based on 2 × 2 contingency tables (infected vs non-infected individuals). *P*-values were adjusted using Holm’s correction for multiple comparisons (six tests per time point), and differences were considered statistically significant when the corrected *P*-value was less than 0.05.

### Analysis of crypt colonization patterns within the symbiotic organ

To examine where bacterial colonization initiates within the M4 symbiotic organ, GFP signals in the crypt lumen were recorded at 12, 16, 20, 24, 32, and 40 h post-inoculation. Five individuals per bacterial strain were dissected and analyzed at each time point (*n* = 5). As shown in Fig. 1A, the crypt-bearing region of the M4 consists of sac-like crypts arranged in two bilaterally symmetric rows along the anterior–posterior axis. Because crypts occur as left–right symmetric pairs, infection status was evaluated at the level of each crypt pair. For each crypt pair, the number of infected crypts (0, 1, or 2) was recorded based on the presence of GFP-positive bacteria in the lumen. For visualization purposes, infection status was expressed as a proportion (0, 0.5, or 1), corresponding to 0/2, 1/2, or 2/2 infected crypts within a pair. To standardize spatial position among individuals with varying total crypt numbers, the relative crypt position was calculated as (crypt pair index)/(total number of crypt pairs in that individual). Crypt pairs were indexed sequentially from the anterior end (M3–M4 junction) toward the posterior end of the M4 region, and relative position values, therefore, ranged from 0 (anterior end) to 1 (posterior end). For statistical analysis, the number of infected crypts per pair (0–2) was modeled using a generalized linear mixed model (GLMM) with a binomial error distribution, specifying the number of infected and uninfected crypts within each pair as the response variable.

### Colonization frequency of crypts at 120 h post-inoculation

At 120 h (5 days) post-inoculation, 10 independent insects were examined for *C. insecticola* WT, *cheA* mutant, and *Pandoraea norimbergensis* each (*n* = 10), whereas *Paraburkholderia fungorum* was examined in nine insects (*n* = 9). For each insect, the number of GFP-positive crypts was counted, and the colonization frequency was calculated as the proportion of colonized crypts relative to the total number of crypts within the M4 region. Each insect was treated as an independent biological replicate for statistical analysis. Comparisons among the four bacterial strains were performed using pairwise Mann–Whitney *U* tests (six pairwise combinations), followed by Holm’s correction for multiple comparisons. Differences were considered statistically significant when the corrected *P*-value was less than 0.05.

### Competitive infection assay in the gut symbiotic organ

Logarithmic-phase cells of the wild-type strain and the *cheA* mutant of *C. insecticola*, together with competitor bacteria (*Paraburkholderia fungorum* or *Pandoraea norimbergensis*), were suspended in sterile distilled water, mixed at a 1:1 ratio, and adjusted to the desired concentrations. For *Pandoraea norimbergensis*, assays were conducted at two inoculation doses (250 CFU/μL and 5,000 CFU/μL). When 250 CFU/μL of *Pandoraea norimbergensis* was used, the focal strain was also adjusted to 250 CFU/μL to maintain equal input ratios. For *Paraburkholderia fungorum*, only 5,000 CFU/μL was used, and the focal strain was adjusted accordingly to 5,000 CFU/μL. For each assay, newly molted second instar nymphs of *R. pedestris* were individually fed 1 μL of the bacterial suspension as described above. The insects were reared at 25°C under long-day conditions with soybean seeds and distilled water. Five days after inoculation, symbiotic organs were dissected from each insect and homogenized in sterile distilled water. The suspensions were serially diluted and plated on YG agar medium containing antibiotics. After 3 days of incubation at 25°C, the colonies of SBE and competitor bacteria were counted. Competitive Index (CI) values were calculated separately for each focal strain to avoid undefined values when one strain was completely excluded from the symbiotic organ. For assays evaluating the *cheA* mutant, CI was calculated as (output *cheA* CFUs/input *cheA* CFUs)/(output competitor CFUs/input competitor CFUs). For assays evaluating wild-type *C. insecticola*, CI was calculated as (output competitor CFUs/input competitor CFUs)/(output *C. insecticola* CFUs/input *C. insecticola* CFUs). CI values were compared against the theoretical value of 1.0 using a two-tailed one-sample *t*-test. Sample sizes were *n* = 10 for all competition conditions except for the *cheA* mutant vs *Paraburkholderia fungorum* assay, for which *n* = 5. Wild-type and chemotaxis mutant strains of *C. insecticola* were selected with rifampicin, and *Paraburkholderia fungorum* and *Pandoraea norimbergensis* were selected with streptomycin. The acquired data were statistically analyzed using the GraphPad Prism 10.1.0 (GraphPad Software).

### Statistics

To understand the spatiotemporal patterns of bacterial infection in M4 crypts, we applied a generalized linear mixed model (GLMM) with a binomial error distribution. The infection ratio was used as the response variable, while the relative crypt position, time, and their interaction were included as fixed effects. Individual ID was incorporated as a random effect. To examine the infection pattern in more detail, we fitted polynomial models of degree *K* and selected the best model for each bacterium based on the AIC. All model coefficients were estimated for each bacterium using the function *glmmTMB()* in the glmmTMB package. To evaluate model fit, we calculated the conditional *R*^2^ following Nakagawa and Schielzeth (2013) using the function *r2()* in the performance package.

Note that we confirmed dispersions of all models using the function *testDispersion()* in the DHARMa package.

## RESULTS

### Strain-dependent spatial localization along the oral–symbiotic organ axis in M3

**Fig 2:**
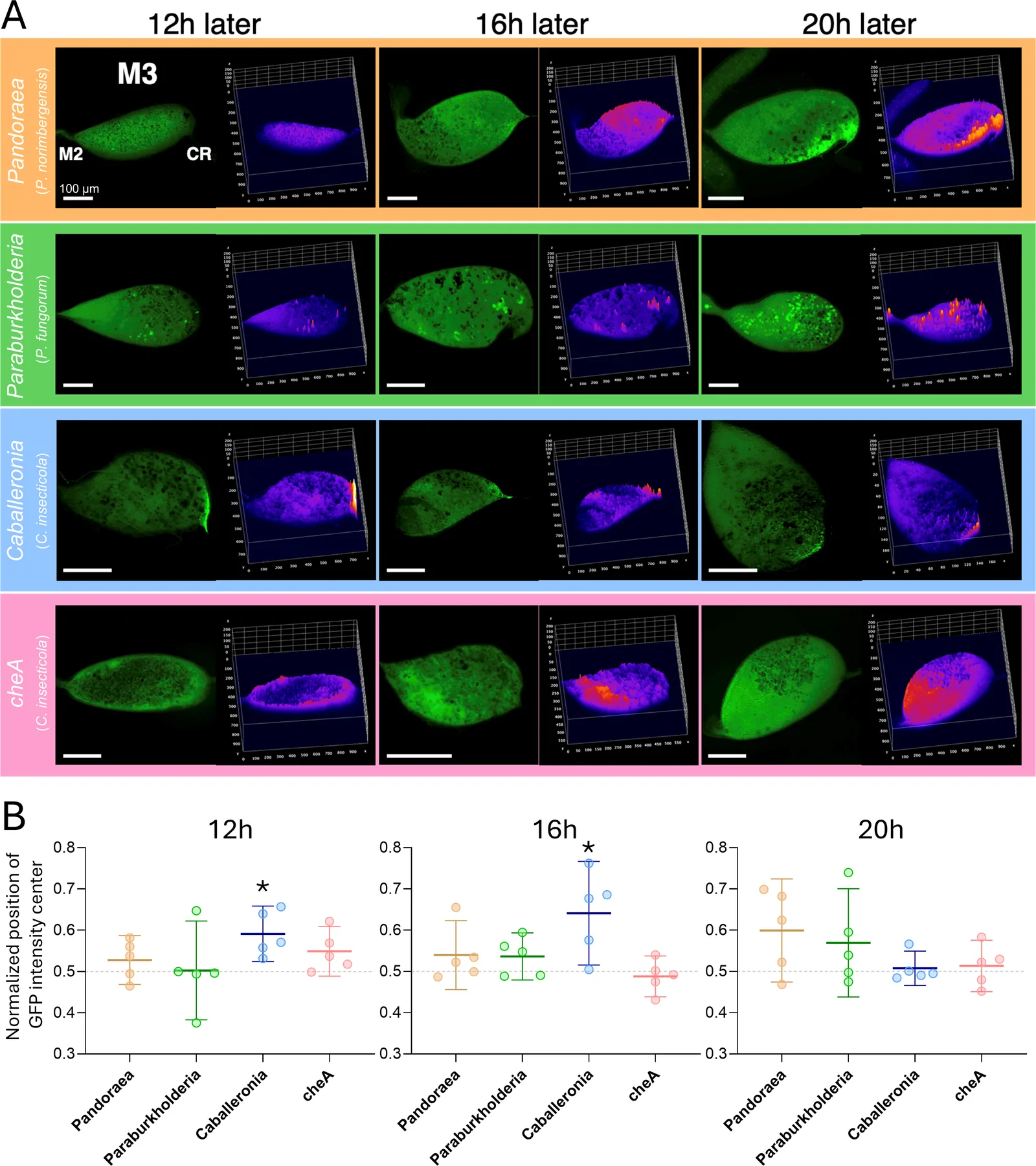
Bacterial distribution in the M3-Constricted region during infection. (**A**) Visualization of bacterial distribution in the M3 region during early infection. Representative images at 12, 16, and 20 h after oral inoculation with GFP-labeled bacteria are shown. For each bacterial strain and each time point, five insects were examined (*n* = 5). In each panel, the left image shows raw GFP fluorescence indicating bacterial localization, and the right image shows a corresponding 3D surface plot generated using ImageJ to visualize the spatial distribution of fluorescence intensity. These plots facilitate visualization of the spatial distribution of GFP fluorescence. Scale bars = 100 µm. (**B**) Quantitative analysis of bacterial spatial distribution in the M3 region. Spatial localization of GFP-expressing bacteria within the M3 region was quantified by calculating the center of mass (COM) of fluorescence intensity along the oral–symbiotic organ axis. Positions were normalized from 0 (oral side) to 1 (symbiotic organ side). A COM value of 0.5 indicates a uniform distribution along the axis, whereas values greater than 0.5 indicate enrichment toward the symbiotic organ side. Dot plots show COM values at 12, 16, and 20 h post inoculation (from left to right). Each dot represents one dissected insect (*n* = 5 per strain at each time point). Horizontal bars indicate the mean, and error bars represent 95% confidence intervals. Statistical significance was evaluated using two-tailed one-sample *t*-tests against a theoretical mean of 0.5. The dashed horizontal line at 0.5 represents the theoretical value expected under a uniform distribution along the oral–symbiotic organ axis. *P < 0.05.

Microscopic observations revealed strain-specific spatial patterns within the M3 region. In individuals orally inoculated with *C. insecticola*, GFP signals were frequently concentrated near the constricted region (CR) at early time points, suggesting directional accumulation toward the symbiotic organ (Fig. 2A and Fig. S2). In contrast, such CR-proximal enrichment was not observed in the *cheA* mutant (Fig. 2A and Fig. S3). In *Pandoraea norimbergensis*, a tendency toward CR-directed localization was observed in some individuals at 16 h and 20 h post-inoculation although this pattern was not consistent across samples (Fig. 2A and Fig. S4). In *Paraburkholderia fungorum*, large GFP-positive aggregates were observed within the M3 lumen, often appearing as prominent clusters rather than showing directional enrichment (Fig. 2A and Fig. S5).

To quantitatively assess bacterial localization along the oral–symbiotic organ axis, spatial distribution was analyzed using center-of-mass (COM) analysis (Fig. S1). COM values greater than 0.5 indicate enrichment toward the symbiotic organ side (Fig. S1). In *C. insecticola*, COM values were significantly greater than 0.5 at 12 h (mean = 0.591, *P* = 0.0198) and 16 h (mean = 0.641, *P* = 0.0354), indicating directional enrichment toward the symbiotic organ side during early time points (Fig. 2B). At 20 h, the COM value was not significantly different from 0.5 (mean = 0.508, *P* = 0.640). In the *cheA* mutant, COM values were not significantly different from 0.5 at any examined time point (12 h: mean = 0.549, *P* = 0.0871; 16 h: mean = 0.488, *P* = 0.545; 20 h: mean = 0.513, *P* = 0.581) (Fig. 2B). Similarly, *Paraburkholderia fungorum* did not show significant deviation from 0.5 at any time point. *Pandoraea norimbergensis* also did not show significant deviation from 0.5 at 12 h, 16 h, or 20 h (20 h: mean = 0.599, *P* = 0.0921) (Fig. 2B). Fluorescence imaging further revealed that in *Paraburkholderia fungorum*, GFP signals were observed as discrete punctate aggregates distributed throughout the M3 region. Together, these results demonstrate that statistically significant directional localization along the oral–symbiotic organ axis was detected only in the wild-type strain at 12 h and 16 h, whereas other strains did not exhibit significant spatial bias under the tested conditions.

### Chemotaxis accelerates early colonization of the M4 symbiotic organ

**Fig 3:**
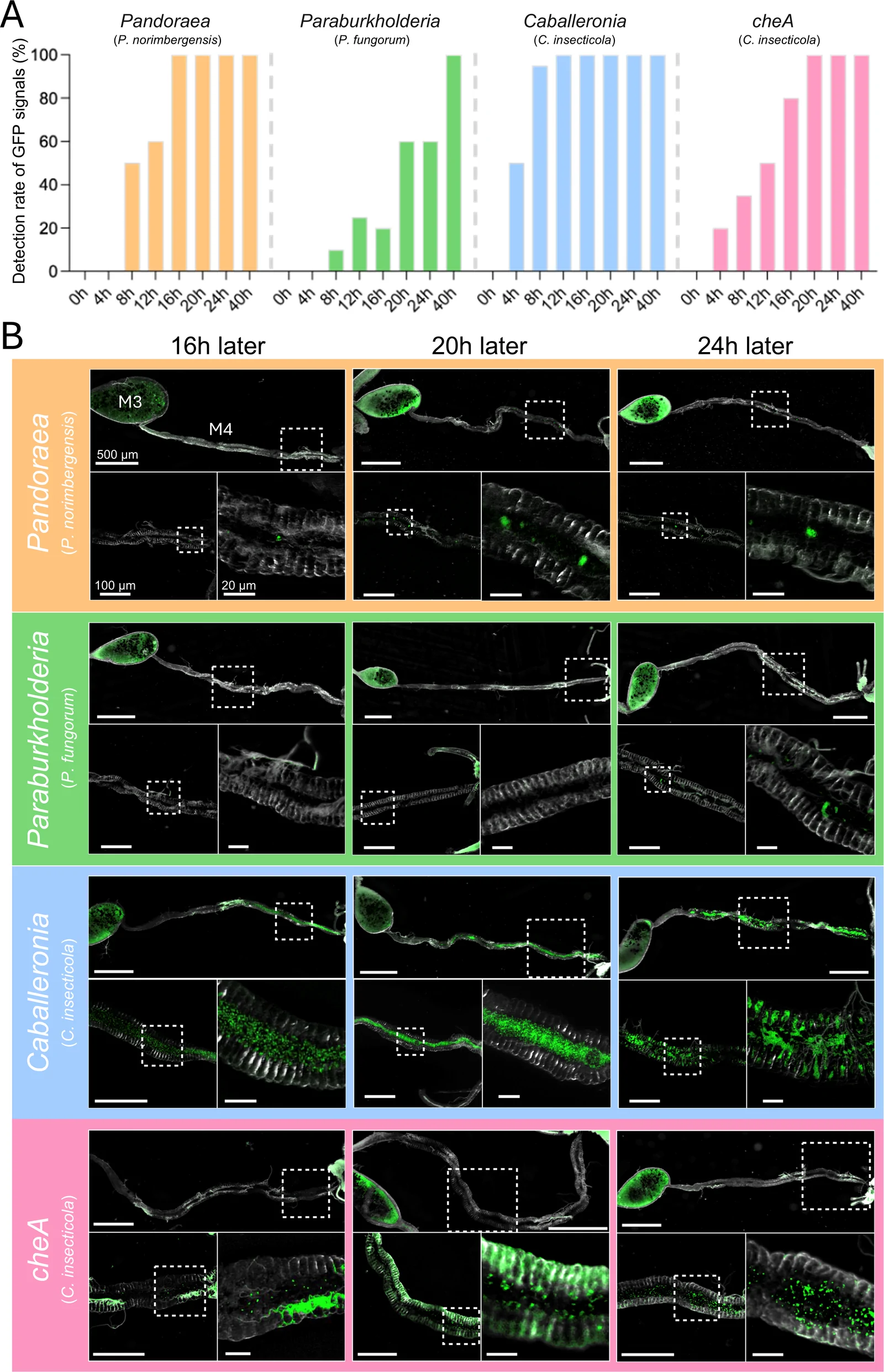
Time to reach the symbiotic organ. (**A**) The time after inoculation at which GFP signals were detected in symbiotic organs. Insects were examined at 0, 4, 8, 12, 16, 20, 24, and 40 h post inoculation. For each time point and each bacterial strain, 20 independent insects were investigated (*n* = 20). If at least one GFP-expressing bacterial cell was detected in the main duct of the M4 region, it was counted as “positive.” The *Y*-axis represents the proportion of positive insects. Detailed statistical comparisons among bacterial strains at each time point are summarized in Fig. S6. (**B**) Representative fluorescence images illustrating the progression of bacterial invasion into the symbiotic organ at 16, 20, and 24 h post-inoculation for each bacterial strain. For each strain and time point, images are arranged to show hierarchical magnification of the same specimen. The upper panels display the entire M3–M4 region (scale bar = 500 µm), with the M3 (digestive region) and M4 (symbiotic region) indicated. White dashed boxes mark areas enlarged in the lower-left panels (scale bar = 100 µm). In these intermediate magnification images, additional dashed boxes indicate regions further enlarged in the lower-right panels (scale bar = 20 µm). GFP fluorescence (green) represents bacterial cells within the M4 region. All images are oriented with the anterior (oral) side to the left and the posterior (hindgut) side to the right.

Second instar nymphs of *R. pedestris* were fed with each bacterial strain, and colonization of the M4 region was monitored (Fig. 3 and Fig. S6). Four hours after inoculation, both wild-type *C. insecticola* and the *cheA* mutant had already begun to colonize the M4 region, whereas *Paraburkholderia fungorum* and *Pandoraea norimbergensis* showed no detectable colonization at this stage (Fig. S6). At 8 h and 12 h post-inoculation, *C. insecticola* had colonized the M4 region in almost all individuals (8h; 95%, 19/20, 12h; 100%, 20/20), and its colonization rate was significantly higher than that of the *cheA* mutant and the two non-native strains (Fisher’s exact test with Holm’s correction, *P* < 0.05, Table S2). In contrast, there was no significant difference between the *cheA* mutant and *Pandoraea* at 8 h–40 h (Fisher’s exact test with Holm’s correction, *P* < 0.05, Table S2). By 16, 20, 24 h, all strains except *Paraburkholderia fungorum* showed significantly higher colonization rates than *Paraburkholderia fungorum* (Fisher’s exact test with Holm’s correction, *P* < 0.05, Table S2). By 40 h, *Paraburkholderia fungorum* also reached 100% colonization. Detailed statistical comparisons among bacterial strains at each time point are provided in Fig. S6. Consistent with these differences in arrival timing, subsequent colonization within the crypts also exhibited strain-specific temporal patterns (Fig. S7). Crypt colonization progressed most rapidly in *C. insecticola* wild type, followed by the *cheA* mutant, whereas *Pandoraea norimbergensis* and *Paraburkholderia fungorum* showed similarly delayed increases in crypt infection frequency. Overall, these results indicate that chemotaxis contributes to accelerated early colonization of the M4 symbiotic organ, as loss of chemotactic function was associated with delayed colonization kinetics.

### Infection patterns after reaching the symbiotic organ

**Fig 4:**
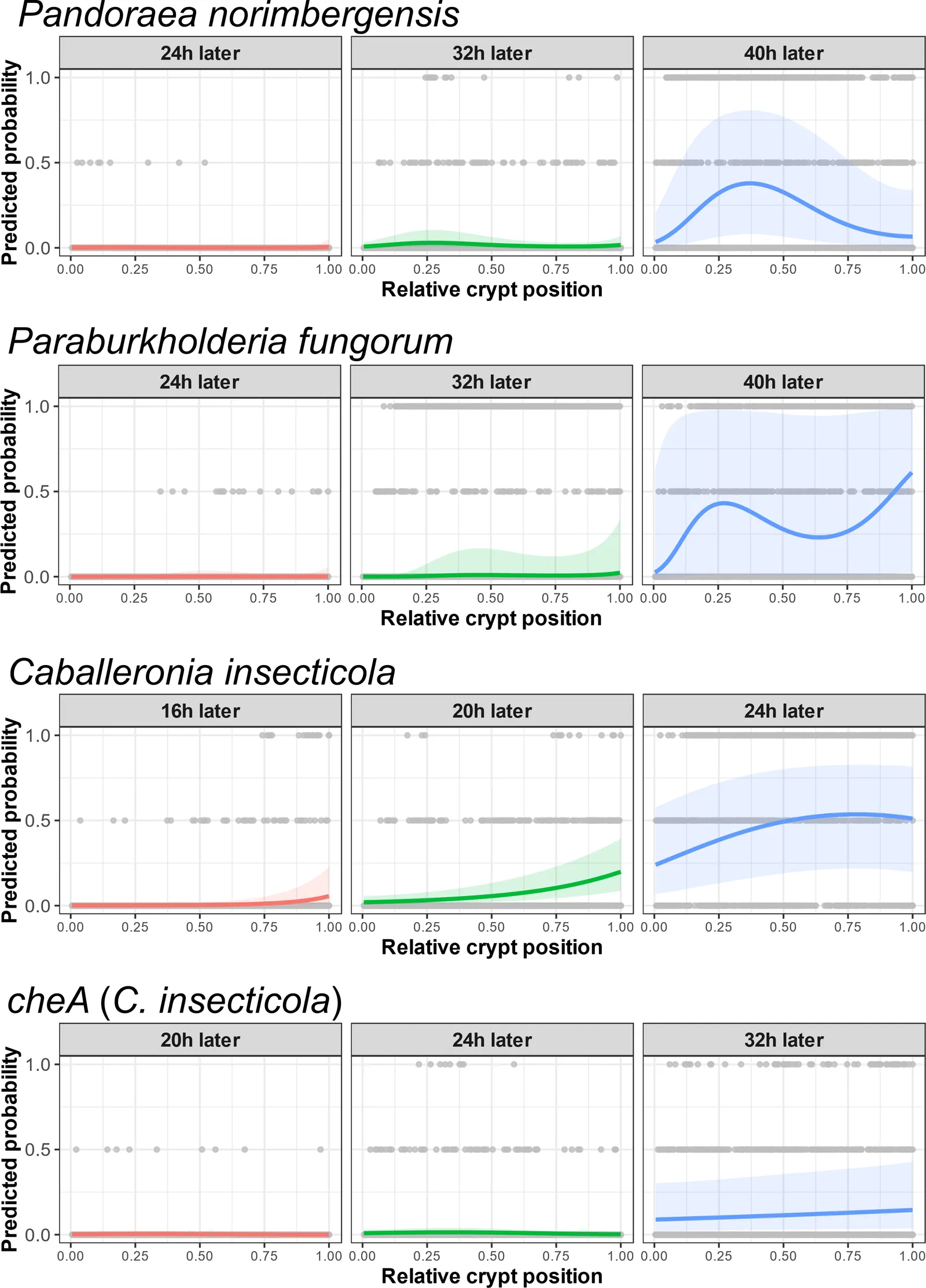
Infection dynamics in crypts after reaching the M4 region. Gray points indicate detected GFP signal of bacteria in each crypt in all individuals. Crypts are arranged in symmetrical pairs; a value of 0.5 indicates that one crypt from the pair was infected, while 1 indicates that both were infected (see main text). *X*-axis indicates the relative crypt position from the M4B side (= 0.00) to the hindgut side (= 1.00). Solid lines and shaded area indicate predictions and 95% CIs from GLMM models, respectively. Different colors mean different observation time.

We first examined the timing of initial crypt colonization based on direct observation of GFP signals. No crypt infection was detected at 12 h post-inoculation for any bacterial strain. Crypt infection by *C. insecticola* was first observed at 16 h (Fig. 4). The *cheA* mutant showed detectable infection from 20 h onward. *Pandoraea norimbergensis* and *Paraburkholderia fungorum* first showed detectable crypt infection at 24 h. GLMM analyses revealed that *C. insecticola* exhibited a posteriorly biased infection probability across crypt positions, and this bias persisted across analyzed time points (Fig. 4). In contrast, the *cheA* mutant did not show a consistent spatial gradient along the crypt axis. Similarly, no posterior bias was detected in *Pandoraea norimbergensis* or *Paraburkholderia fungorum* (Fig. 4). Together, these results indicate that chemotaxis contributes not only to accelerated colonization but also to the establishment of a spatially biased infection pattern within the M4 organ.

Five days post-inoculation, both wild-type and *cheA* mutant strains had colonized most crypts (Fig. S8). Colonization frequency of these strains was significantly higher than that of *Pandoraea norimbergensis* and *Paraburkholderia fungorum* (Mann-Whitney *U* test with Holm’s correction, *P* < 0.05, wild-type *C. insecticola* vs *cheA*, corrected *P* = 0.8885; wild-type *C. insecticola* vs *Paraburkholderia fungorum*, corrected *P* = 0.0244; wild-type *C. insecticola* vs *Pandoraea norimbergensis*, corrected *P* < 0.0006; *cheA* vs *Paraburkholderia fungorum*, corrected *P* = 0.0426; *cheA* vs *Pandoraea norimbergensis*, corrected *P* < 0.0006; *Paraburkholderia fungorum* vs *Pandoraea norimbergensis*, corrected *P* = 0.0558). These results suggest that chemotaxis is dispensable for final crypt occupancy but influences early spatial dynamics, whereas *Pandoraea norimbergensis* remains less efficient in overall crypt colonization.

### Importance of chemotaxis in *in vivo* competition

**Fig 5:**
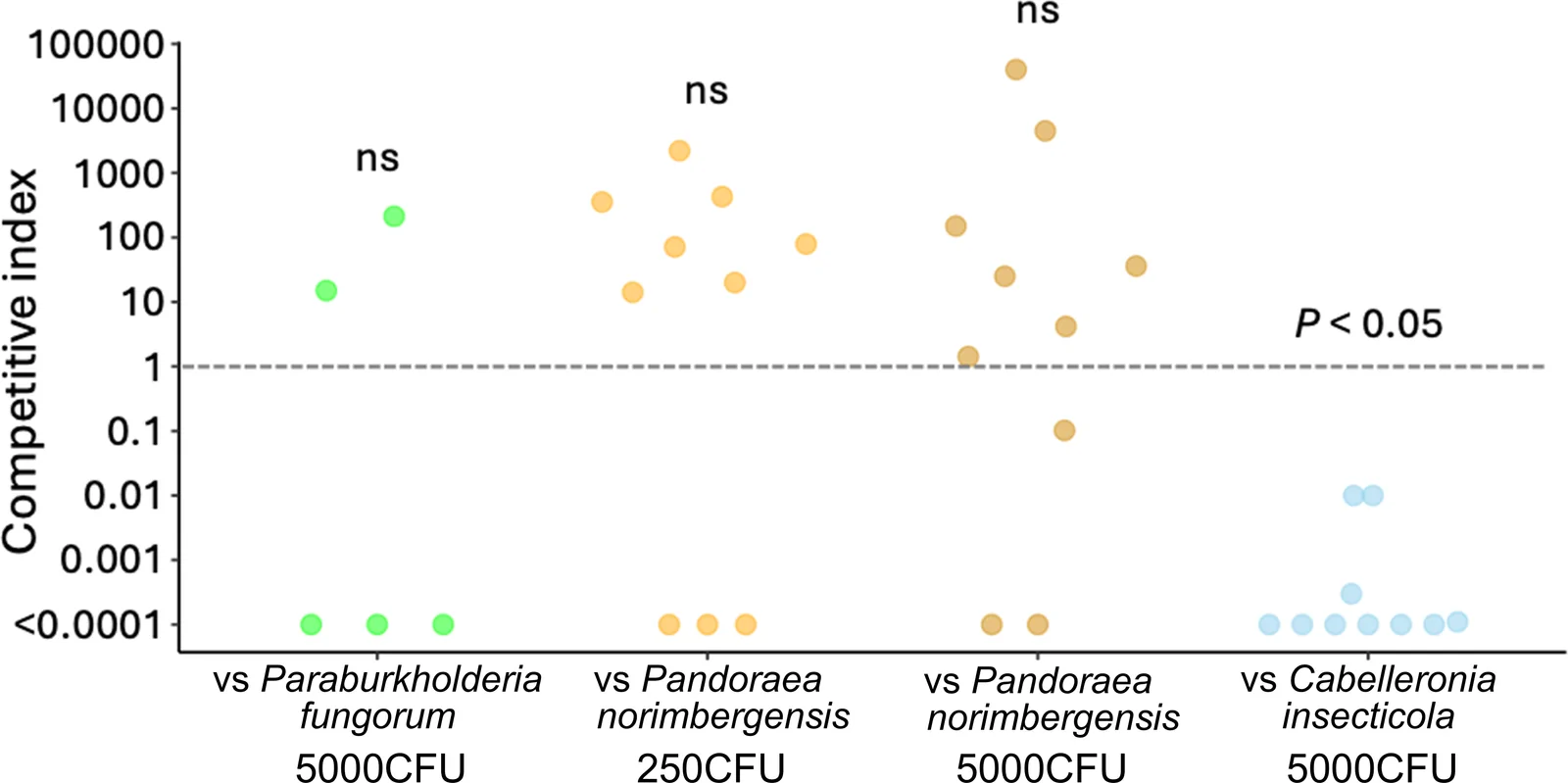
Competitiveness of chemotaxis mutant. Competitive index (CI) values of the *cheA* mutant within symbiotic organs were calculated as (output *cheA* CFU/input *cheA* CFU) divided by (output competitor CFU/input competitor CFU). The *x*-axis indicates the competitor strain used in each co-inoculation assay, and the number shown beneath each strain name represents the colony-forming units (CFU) of both strains orally administered per insect. Unless otherwise indicated, 5,000 CFU of the *cheA* mutant and 5,000 CFU of the competitor strain were co-inoculated. For *Pandoraea norimbergensis*, competitions were performed using either 250 CFU or 5,000 CFU per strain; when 250 CFU of *Pandoraea norimbergensis* was used, the *cheA* mutant was also administered at 250 CFU to maintain equal input ratios. CI values were compared against the theoretical value of 1.0 using a two-tailed one-sample *t*-test. CI > 1 indicates that the *cheA* mutant outcompeted the co-inoculated strain, whereas CI < 1 indicates a competitive disadvantage. Each dot represents an individual insect. Sample size was *n* = 10 per condition unless otherwise indicated; the *cheA* mutant vs *Paraburkholderia fungorum* condition was performed with *n* = 5. The dashed horizontal line indicates the theoretical CI value of 1.0 used as the reference value for the one-sample *t*-test. ns, not significant (*P* ≥ 0.05).

When wild-type *C. insecticola* was co-inoculated with competitors, competitive dominance was observed against *Paraburkholderia fungorum* at 5,000 CFU/μL and against *Pandoraea norimbergensis* at 250 CFU/μL (1-sample *t* test, *P* < 0.05) (Fig. S9). However, dominance over *Pandoraea norimbergensis* was not detected at 5,000 CFU/μL. Under conditions where the wild type showed competitive dominance, the *cheA* mutant failed to achieve dominance (Fig. 5). Furthermore, direct competition between wild type and *cheA* revealed significant dominance of the wild type (*P* < 0.05) (Fig. 5). These results demonstrate that chemotaxis enhances competitive fitness within the symbiotic organ under conditions of direct competition.

## DISCUSSION

Host-microbe symbioses without vertical transmission are characterized by multiple-layered partner choice mechanisms that facilitate the establishment of specific relationships (1). In the well-known legume-rhizobia symbiosis, chemical communication between the host and microbe via nod factors is crucial, and bacterial motility and chemotaxis are important for rapid infection and the formation of specific symbioses (18, 19). Similarly, in the Hawaiian bobtail squid*—Aliivibrio fischeri* symbiosis, chemotaxis toward host-derived chitobiose enables symbionts to navigate into the light organ and is essential for successful early colonization (20, 21). Together, these examples suggest that chemotaxis commonly facilitates rapid establishment of environmentally acquired mutualistic symbioses. In the bean bug *Riptortus pedestris*, once *Caballeronia insecticola* colonizes the M4 symbiotic organ, the CR undergoes closure over the subsequent 12–18 h, which is associated with the establishment of resistance to secondary infection; importantly, bacteria introduced after the initial infection can still colonize the M4 during an early period but are unable to establish infection at later time points (14). Thus, resistance to secondary infection appears to be established over time following the initial colonization of the M4. In this study, we found significant differences in the time required for bacteria to reach the symbiotic organ, with *C. insecticola* arriving more rapidly than *Paraburkholderia fungorum* and *Pandoraea norimbergensis* (Fig. 3 and Fig. S6). Importantly, directional enrichment of *C. insecticola* toward the CR was already detectable within the M3 region during early time points, whereas this spatial bias was not observed in the chemotaxis-deficient mutant (Fig. 2 and Fig. S1 and S2). These findings suggest that chemotaxis may facilitate efficient navigation through the M3 region toward the symbiotic organ shortly after inoculation. Furthermore, a *cheA* mutant of *C. insecticola* exhibited delayed arrival compared to the wild-type strain, indicating that chemotaxis contributes to rapid colonization of the symbiotic organ. Although both strains ultimately achieved comparable levels of crypt colonization after 5 days, co-infection experiments demonstrated reduced competitive performance of the chemotaxis mutant within the organ (Fig. 5 and Fig. S9). One possible explanation is that the rate at which bacteria reach the symbiotic organ may also influence the number of cells that initially establish within the M4. More rapid arrival could allow *C. insecticola* to establish a larger initial population before complete closure of the CR, thereby providing an advantage in subsequent within-organ competition. Conversely, delayed arrival caused by impaired chemotaxis may reduce the initial population size and thereby compromise competitive performance, even if the mutant can eventually achieve colonization levels comparable to those of the wild type. Together, these results suggest that chemotaxis accelerates early colonization and may contribute to competitive success by promoting earlier establishment within the symbiotic organ.

Another notable finding of this study is that colonization within the symbiotic organ exhibited strain-specific spatial patterns. Among the tested strains, only *C. insecticola*, which showed superior competitive performance within the organ, displayed a pronounced posterior bias in crypt infection (Fig. 4). In contrast, neither the chemotaxis mutant nor the non-native strains exhibited a posterior bias in crypt colonization. After colonization and proliferation within the crypts, a portion of the symbiont population is transported to the anterior M4B region, where bacteria are digested and absorbed by the host (22). In this context, preferential infection of posterior crypts could potentially delay the transit of bacteria toward the digestive M4B region, thereby prolonging residence time within the crypts and enhancing competitive persistence. Although this possibility remains speculative, it suggests that spatial positioning within M4 may influence intra-organ competitive dynamics. In addition to such within-organ ecological effects, posterior crypts play specialized roles in some stinkbug species (23). For example, in the brown-winged green stinkbug, posterior crypts of the adult symbiotic organ become hypertrophied compared to other crypts (24, 25), and symbionts residing in these crypts are applied to the egg surface (26, 27), ensuring vertical transmission. In several stinkbugs associated with *Caballeronia*, vertical transmission via eggs or adult feces has also been documented (28, 29). Although the functional consequences of posteriorly biased colonization remain unclear in *R. pedestris*, these observations raise the possibility that spatial infection patterns within M4 may influence symbiont persistence, competitive stability, or transmission potential. Further experimental work will be required to determine the adaptive significance of this spatial bias.

Microscopic observations further revealed strain-specific differences in bacterial aggregation patterns within the symbiotic organ. *Paraburkholderia fungorum* frequently formed aggregates in M3 (Fig. S5) and appeared as dispersed cells or short chains in M4 (Fig. S10), whereas *Pandoraea norimbergensis* formed distinct clusters within the main duct of M4 (Fig. 3 and Fig. S10). In contrast, *C. insecticola* cells were generally dispersed in both M3 and M4 and did not form comparable aggregates (Fig. 3 and Fig. S2 and S10). These observations suggest that aggregation process differs among bacterial species and may represent an additional layer of variation in colonization strategies within the host gut. The factors underlying these differences remain unclear. However, it is possible that host-derived antimicrobial peptides (AMPs) and bacterial biofilm-forming tendencies may be related to these aggregation patterns (30–32). Given that AMP expression levels vary along the gut regions (6, 33), differences in sensitivity to host immune factors could potentially influence whether bacteria appear as aggregates or remain dispersed. These possibilities remain speculative and warrant further investigation.

An important question arising from the present study is the identity of the host-derived cues that guide *C. insecticola* toward the M4 symbiotic organ. In the squid–Aliivibrio fischeri symbiosis, host-derived chitobiose functions as a chemoattractant that directs symbionts through the light-organ ducts into the crypts, whereas many pathogenic bacteria similarly exploit host-derived metabolites or signaling molecules to navigate toward favorable colonization sites. Our observation that wild-type *C. insecticola* exhibited directional enrichment toward the constricted region (CR) within the M3 region suggests that host-derived chemoattractants may guide bacteria to the entrance of the symbiotic organ. Moreover, the preferential colonization of posterior crypts raises the intriguing possibility that additional spatial cues operate within the M4 organ itself, establishing the microbiogeography of symbiont colonization after entry into the organ. Identification of these putative host-derived signals will be an important goal for future studies and may reveal how hosts actively organize the spatial distribution of beneficial symbionts within specialized symbiotic organs.

Collectively, our findings indicate that chemotaxis accelerates early colonization of the M4 symbiotic organ and enhances competitive performance *in vivo*. Although chemotaxis is not essential for eventual crypt colonization, rapid arrival likely provides a substantial ecological advantage during the initial stages of symbiont establishment. These results highlight the importance of spatial and temporal dynamics in shaping symbiotic associations.

## Data Availability

All data supporting the findings of this study, including the data underlying the figures and statistical analyses, are publicly available in Figshare at https://doi.org/10.6084/m9.figshare.33268368.

## SUPPLEMENTAL MATERIAL

Supplemental material aem.01364-26-s0001.docx Fig. S1 to 10; Tables S1 and S2.

ASM does not own the copyrights to Supplemental Material that may be linked to, or accessed through, an article. The authors have granted ASM a non-exclusive, world-wide license to publish the Supplemental Material files. Please contact the corresponding author directly for reuse.
