# Supplementary material for: Chemotaxis promotes early colonization and microbiogeography of Caballeronia insecticola in the gut symbiotic organ of Riptortus pedestris
Source: Appl Environ Microbiol. 2026 Sep 14;92(10):e01364-26. doi: 10.1128/aem.01364-26 (PMC13689054; doi:10.1128/aem.01364-26)
Supplement: Supplementary file 1 [file aem.01364-26-s0001.docx]

FIGURE S1

**FIGURE S1. Definition of the M3 measurement axis and fluorescence intensity profiling.**

(A) Representative GFP fluorescence image of the M3 region following oral inoculation. A straight line (white line) was drawn from the anterior entrance of the M3 region (junction with M2) to the constricted region (CR), along which fluorescence intensity was quantified. The CR, corresponding to the entrance of the symbiotic organ, is indicated by an arrowhead. This line defines the oral–symbiotic organ axis used for subsequent analyses. (B) Fluorescence intensity profile extracted along the measurement axis shown in (A). The x-axis represents the normalized position along the M3 region, where 0 corresponds to the oral side and 1 corresponds to the symbiotic organ (CR) side. The y-axis indicates background-corrected fluorescence intensity. These intensity distributions were used to calculate the center of mass (COM) of fluorescence for each sample. The dashed gray line indicates the calculated center of mass (COM) of the fluorescence intensity distribution, representing the weighted average position of the signal.

FIGURE S2

**FIGURE S2. GFP localization of *Caballeronia insecticola* within the M3 region at early time points after oral** inoculation**.**

Representative fluorescence microscopy images of the M3 region at 12, 16, and 20 h post-inoculation. GFP signals (green) indicate bacterial localization within the gut lumen. Images are representative of n = 5 individuals per time point.

FIGURE S3

**FIGURE S3. GFP localization of the *cheA* mutant of *Caballeronia insecticola* within the M3 region after oral** inoculation**.**

Representative fluorescence microscopy images of the M3 region at 12, 16, and 20 h post-inoculation. GFP signals (green) indicate bacterial localization within the gut lumen. Images are representative of n = 5 individuals per time point.

FIGURE S4

**FIGURE S4. GFP localization of *Pandoraea norimbergensis* within the M3 region after oral** inoculation**.**

Representative fluorescence microscopy images of the M3 region at 12, 16, and 20 h post-inoculation. GFP signals (green) indicate bacterial localization within the gut lumen. Images are representative of n = 5 individuals per time point.

FIGURE S5

**FIGURE S5. GFP localization of *Paraburkholderia fungorum* within the M3 region after oral** inoculation**.**

Representative fluorescence microscopy images of the M3 region at 12, 16, and 20 h post-inoculation. GFP signals (green) indicate bacterial localization within the gut lumen. Images are representative of n = 5 individuals per time point.

FIGURE S6

**FIGURE S6. Statistical comparison of infection rates among bacterial strains at each time point.**

The proportion of insects in which GFP signals were detected in the M4 region is shown for each bacterial strain at each time point. For each time point and each bacterium, twenty insects were examined (n = 20). An individual was scored as positive if at least one GFP-expressing bacterium was detected in the main duct of the M4 region. For each time point, infection rates among the four bacterial strains were compared using pairwise Fisher’s exact tests (2×2 contingency tables; six comparisons per time point), followed by Holm's correction. Different letters indicate statistically significant differences among bacterial strains within the same time point (corrected P < 0.05). Bars sharing the same letter are not significantly different.

FIGURE S7

**FIGURE S7. Time-course of crypt colonization frequency for each bacterial strain.**

The proportion of infected crypts within the M4 symbiotic organ was quantified at multiple time points after oral inoculation. Crypts with detectable GFP signals in the lumen were considered positive. Each panel represents a different bacterial strain: (A) *Pandoraea norimbergensis*, (B) *Paraburkholderia fungorum*, (C) *Caballeronia insecticola* wild type (WT), and (D) *C. insecticola* *cheA* mutant. At each time point, five independent insects were examined per strain (n = 5).

FIGURE S8

**FIGURE S8. Colonization frequency of crypts 5 days after oral inoculation.**

(A) Images of the GFP-labeled bacteria in the symbiotic organ. (B) Colonization frequency of crypts at 120 h post-inoculation. Each data point represents one independent insect (*C. insecticola*, n = 10; *cheA* mutant, n = 10; *Pandoraea norimbergensis*, n = 10; *Paraburkholderia fungorum*, n = 9). The proportion of colonized crypts was calculated for each individual. Statistical comparisons among the four bacterial strains were performed using pairwise Mann–Whitney U tests (six comparisons), followed by Holm's correction. Different letters indicate statistically significant differences among bacterial strains (corrected P < 0.05). Groups sharing the same letter are not significantly different. Horizontal bars indicate medians.

FIGURE S9

**FIGURE S9. Competitiveness of *Caballeronia insecticola*.**

Competitive index (CI) values of wild-type *C. insecticola* within symbiotic organs were calculated as (output competitor CFU / input competitor CFU) divided by (output *C. insecticola* CFU / input *C. insecticola* CFU). The x-axis indicates the competitor strain used in each co-inoculation assay, and the number shown beneath each strain name represents the CFU of both strains orally administered per insect. Unless otherwise indicated, 5,000 CFU of *C. insecticola* and 5,000 CFU of the competitor strain were co-inoculated. For *Pandoraea norimbergensis*, assays were performed using either 250 CFU or 5,000 CFU per strain; when 250 CFU of *Pandoraea norimbergensis* was administered, *C. insecticola* was also inoculated at 250 CFU to maintain equal input ratios. CI values were compared against 1.0 using a two-tailed one-sample t-test. CI > 1 indicates that the competitor strain outcompeted *C. insecticola*, whereas CI < 1 indicates that *C. insecticola* was competitively superior. Each dot represents an individual insect (n = 10 per condition). The dashed horizontal line indicates the theoretical CI value of 1.0 (null hypothesis) used for the two-tailed one-sample t-test.

FIGURE S10

FIGURE S10. GFP-labeled bacterial cells within the main duct of the M4 symbiotic organ at 40 h post-oral inoculation.

Representative fluorescence micrographs of GFP-expressing *Caballeronia* *insecticola*, *Pandoraea* *norimbergensis*, and *Paraburkholderia fungorum* within the main duct of the M4 symbiotic organ at 40 h after oral infection. Bacterial cells are shown in green. Yellow arrowheads indicate examples of clustered bacterial cells observed within the main duct.

Table S1. Strains and mutants used in this study

Kikuchi Y., & Fukatsu T. (2014). Live imaging of symbiosis: spatiotemporal infection dynamics of a GFP‐labelled *Burkholderia* symbiont in the bean bug *Riptortus pedestris*. *Molecular Ecology* 23(6), 1445–1456.

Lachat J. et al. (2024). Hundreds of antimicrobial peptides create a selective barrier for insect gut symbionts. *PNAS*, 121(25), 2017.

Ohbayashi T. et al. (2015). Insect’s intestinal organ for symbiont sorting. *PNAS*, 112(37), E5179–E5188.

Itoh H. et al. (2019). Host–symbiont specificity determined by microbe–microbe competition in an insect gut. *PNAS* 116(45), 22673–22682.

Table S2. Pairwise comparisons of colonization rates among bacterial strains at each time point
